# Supplementary material for: Exogenous Melatonin Reinforces Photosynthesis, Antioxidant Defense and Gene Expression to Ameliorate Na2CO3 Stress in Maize
Source: Plants (Basel). 2024 Oct 11;13(20):2844. doi: 10.3390/plants13202844 (PMC11511503; doi:10.3390/plants13202844)
Supplement: Supplementary file 1 [file plants-13-02844-s001.zip › plants-3194664-supplementary.pdf]

Table S1. Multiple factorial analysis of variance (ANOVA) of the three examined variables (maize genotypes, melatonin (MT) application, and Na2CO3 stress), and their interactions on 25 tested traits in 2 maize genotype seedlings under different treatments.

Trait	Genotypes (G)	MT application (M)	Na_2CO_3 stress (N)	G × M Interaction	G × N Interaction	M × N Interaction	G × M × N Interaction
Growth parameter							
SW	F=293.846 ($p < 0.01$)	F=11.613 ($p < 0.05$)	F=99.567 ($p < 0.01$)	F=0.003 ($p > 0.05$)	F=2.867 ($p > 0.05$)	F=0.626 ($p > 0.05$)	F=0.498 ($p > 0.05$)
RW	F=102.119 ($p < 0.01$)	F=8.094 ($p < 0.05$)	F=81.9 ($p < 0.01$)	F=0.322 ($p > 0.05$)	F=3.575 ($p > 0.05$)	F=1.358 ($p > 0.05$)	F=0.219 ($p > 0.05$)
SL	F=117.896 ($p < 0.01$)	F=18.999 ($p < 0.01$)	F=84.356 ($p < 0.01$)	F=5.095 ($p < 0.05$)	F=1.551 ($p > 0.05$)	F=4.254 ($p > 0.05$)	F=0.341 ($p > 0.05$)
SA	F=86.728 ($p < 0.01$)	F=16.071 ($p < 0.01$)	F=59.154 ($p < 0.01$)	F=0.822 ($p > 0.05$)	F=0.310 ($p > 0.05$)	F=5.426 ($p < 0.05$)	F=0.381 ($p > 0.05$)
Photosynthetic performance							
Chl a	F=1526.223 ($p < 0.01$)	F=234.472 ($p < 0.01$)	F=921.329 ($p < 0.01$)	F=11.266 ($p < 0.05$)	F=15.303 ($p < 0.05$)	F=4.887 ($p < 0.05$)	F=8.161 ($p < 0.05$)
Chl b	F=881.143 ($p < 0.01$)	F=115.734 ($p < 0.01$)	F=318.597 ($p < 0.01$)	F=9.448 ($p < 0.01$)	F=13.545 ($p < 0.01$)	F=10.185 ($p < 0.01$)	F=3.864 ($p > 0.05$)
Car	F=10617.084 ($p < 0.01$)	F=398.44 ($p < 0.01$)	F=7020.877 ($p < 0.01$)	F=11.972 ($p < 0.01$)	F=2286.435 ($p < 0.01$)	F=95.874 ($p < 0.01$)	F=25.402 ($p < 0.01$)
Chl a/b	F=2231.112 ($p < 0.01$)	F=329.807 ($p < 0.01$)	F=1197.072 ($p < 0.01$)	F=3.212 ($p > 0.05$)	F=25.15 ($p < 0.01$)	F=10.771 ($p < 0.01$)	F=11.366 ($p < 0.01$)
Pn	F=1769.026 ($p < 0.01$)	F=114.864 ($p < 0.01$)	F=3185.756 ($p < 0.01$)	F=6.570 ($p < 0.05$)	F=46.34 ($p < 0.01$)	F=140.736 ($p < 0.01$)	F=39.46 ($p < 0.01$)
Ci	F=607.036 ($p < 0.01$)	F=148.467 ($p < 0.01$)	F=3686.901 ($p < 0.01$)	F=31.929 ($p > 0.05$)	F=0.051 ($p < 0.01$)	F=242.723 ($p < 0.01$)	F=15.881 ($p < 0.01$)
Gs	F=2046.216 ($p < 0.01$)	F=656.639 ($p < 0.01$)	F=3607.745 ($p < 0.01$)	F=5.205 ($p < 0.05$)	F=126.93 ($p < 0.01$)	F=915.369 ($p < 0.01$)	F=156.986 ($p < 0.01$)
Tr	F=10224.121 ($p < 0.01$)	F=10506.233 ($p < 0.01$)	F=72188.349 ($p < 0.01$)	F=444.097 ($p < 0.01$)	F=20.843 ($p < 0.01$)	F=5111.278 ($p < 0.01$)	F=2.361 ($p > 0.05$)
Membrane characteristics							
REC	F=111.259 ($p < 0.01$)	F=11.732 ($p < 0.05$)	F=150.98 ($p < 0.01$)	F=2.978 ($p > 0.05$)	F=14.421 ($p < 0.05$)	F=9.006 ($p > 0.05$)	F=2.38 ($p > 0.05$)
ROS level							
H_2O_2	F=0.973 ($p > 0.05$)	F=687.669 ($p < 0.01$)	F=1734.042 ($p < 0.01$)	F=1.590 ($p > 0.05$)	F=3.304 ($p > 0.05$)	F=254.762 ($p < 0.01$)	F=0.015 ($p > 0.05$)
Antioxidant enzymes activity							
SOD	F=83.512 ($p < 0.01$)	F=117.514 ($p < 0.01$)	F=1093.202 ($p < 0.01$)	F=2.202 ($p > 0.05$)	F=1.121 ($p > 0.05$)	F=83.542 ($p < 0.01$)	F=42.627 ($p < 0.01$)
POD	F=40.6 ($p < 0.01$)	F=5.576 ($p < 0.05$)	F=96.68 ($p < 0.01$)	F=0.002 ($p > 0.05$)	F=2.089 ($p > 0.05$)	F=2.924 ($p > 0.05$)	F=0.424 ($p > 0.05$)
CAT	F=73.32	F=66.286	F=1342.286	F=0.493	F=66.286	F=0.966	F=22.778

	($p < 0.01$)	($p < 0.01$)	($p < 0.01$)	($p > 0.05$)	($p < 0.01$)	($p > 0.05$)	($p < 0.01$)
APX	F=89.308	F=89.308	F=1231.482	F=5.511	F=64.091	F=39.946	F=0.438
	($p < 0.01$)	($p < 0.01$)	($p < 0.01$)	($p < 0.05$)	($p < 0.01$)	($p < 0.01$)	($p > 0.05$)
Cell osmotic pressure							
RWC	F=71.781	F=13.459	F=120.623	F=5.105	F=11.782	F=10.607	F=4.819
	($p < 0.01$)	($p < 0.05$)	($p < 0.01$)	($p < 0.05$)	($p < 0.05$)	($p < 0.05$)	($p < 0.05$)
stomatal morphology							
PSL	F=2284.202	F=92.877	F=478.062	F=5.864	F=1.873	F=2.111	F=40.174
	($p < 0.01$)	($p < 0.01$)	($p < 0.01$)	($p < 0.05$)	($p > 0.05$)	($p > 0.05$)	($p < 0.01$)
DSL	F=409.549	F=365.623	F=269.314	F=63.733	F=94.369	F=6.45	F=2.092
	($p < 0.01$)	($p < 0.01$)	($p < 0.01$)	($p < 0.01$)	($p < 0.01$)	($p < 0.05$)	($p > 0.05$)
PSW	F=7.348	F=32.125	F=71.911	F=0.131	F=0.093	F=4.791	F=0.131
	($p < 0.05$)	($p < 0.01$)	($p < 0.01$)	($p > 0.05$)	($p > 0.05$)	($p < 0.05$)	($p > 0.05$)
DSW	F=2079.043	F=278.294	F=1382.348	F=6.591	F=2.25	F=83.75	F=46.181
	($p < 0.01$)	($p < 0.01$)	($p < 0.01$)	($p < 0.05$)	($p > 0.05$)	($p < 0.01$)	($p < 0.01$)
PSA	F=1665.708	F=65.615	F=431.921	F=0.455	F=0.178	F=0.011	F=4.818
	($p < 0.01$)	($p < 0.01$)	($p < 0.01$)	($p > 0.05$)	($p > 0.05$)	($p > 0.05$)	($p < 0.05$)
DSA	F=1264.801	F=135.369	F=290.6	F=2.98	F=5.316	F=14.257	F=13.409
	($p < 0.01$)	($p < 0.01$)	($p < 0.01$)	($p < 0.05$)	($p < 0.05$)	($p < 0.01$)	($p < 0.01$)

SW, seedling weight; RW, root weight; SL, seedling length; LA, leaf area; Chl a, chlorophyll a content; Chl b, chlorophyll b content; Car, carotenoid content; Chl a/b, chlorophyll a/b; Pn, net photosynthetic rate; Ci, intercellular CO_2 concentration; Gs, stomatal conductance; Tr, transpiration rate; REC, relative electrical conductivity; ROS, reactive oxygen species; H_2O_2, H_2O_2 content; SOD, superoxide dismutase activity; POD, peroxidase activity; CAT, catalase activity; APX, ascorbate peroxidase activity; RWC, relative water content; PSL, paraxial surface stomatal length; DSL, dorsal surface stomatal length; PSW, paraxial surface stomatal width; DSW, dorsal surface stomatal width; PSA, paraxial surface stomatal area; DSA, dorsal surface stomatal area.
